# Persistent impairments 3 years after (neo)adjuvant chemotherapy for breast cancer: results from the MaTox project

**DOI:** 10.1007/s10549-017-4365-7

**Published:** 2017-07-05

**Authors:** Hans-Jürgen Hurtz, Hans Tesch, Thomas Göhler, Ulrich Hutzschenreuter, Johanna Harde, Lisa Kruggel, Martina Jänicke, Norbert Marschner, TMK-Group (Tumour Registry Breast Cancer)

**Affiliations:** 1 Joint Outpatient-Centre for Oncology, Niemeyerstr. 22, 06110 Halle (Saale), Germany; 2 Outpatient-Centre for Haematology and Medical Oncology at Bethanien, Im Prüfling 17-19, 60389 Frankfurt am Main, Germany; 3 Onkozentrum Dresden, Leipziger Str. 118, 01127 Dresden, Germany; 4 Joint Outpatient-Centre for Haematology and Oncology, Albert-Schweitzer-Str. 30, 48527 Nordhorn, Germany; 5 Statistics, iOMEDICO, Hanferstr. 28, 79108 Freiburg, Germany; 6 Clinical Epidemiology and Health Economics, iOMEDICO, Hanferstr. 28, 79108 Freiburg, Germany; 7 Outpatient-Centre for Interdisciplinary Oncology and Haematology, Wirthstr. 11c, 79110 Freiburg, Germany

**Keywords:** Breast neoplasms, Chemotherapy, adjuvant, Cohort studies, Drug-related side effects and adverse reactions, Outpatients, Questionnaires

## Abstract

**Purpose:**

Although treatment for early breast cancer improved prognosis greatly, it can have significant long-term consequences, which must be considered during treatment decision.

**Methods:**

453 patients with neoadjuvant or adjuvant treatment intention were recruited into the MaTox project within the prospective, multicentre, population-based German TMK cohort study (Tumour Registry Breast Cancer) between 2008 and 2009. Patient-reported outcomes (PROs) on 26 treatment-related symptoms were assessed via a specifically designed questionnaire at 4 weeks, 6 months, 18 months and 3 years after start of systemic treatment.

**Results:**

The results show that alterations in smell, taste and appetite were clearly improved 3 years after treatment. In contrast, post-surgical symptoms, restrictions in memory/attention, musculoskeletal system and polyneuropathy worsened substantially over time and were persistent after 3 years: 78% of the patients recorded impairment in memory, 73% muscle pain, 67% pain at the operated site and 57% paraesthesia in fingers or toes. A logistic regression model showed that risk factors for developing persistent paraesthesia symptoms were age, early paraesthesia symptoms and taxane-based therapy.

**Conclusions:**

Our data show that most patients with breast cancer have persistent impairments negatively influencing their daily life even 3 years after treatment. Furthermore, we highlight areas requiring special attention in follow-up care.

**Electronic supplementary material:**

The online version of this article (doi:10.1007/s10549-017-4365-7) contains supplementary material, which is available to authorized users.

## Introduction

In women, breast cancer is the most common cancer in the world and the second most common cause of cancer death after lung cancer in more developed regions [1]. In Germany, about 70,000 women are diagnosed with breast cancer every year, the majority of them with early breast cancer [2]. Patients with localized, operable breast cancer usually have a good prognosis, with a relative 5-year survival rate of up to 90% [3]. The ten-year survival exceeds 70% in most European regions [4, 5]. Increased use of (neo)adjuvant systemic treatment has considerably improved patients‘ survival [6, 7] resulting in more breast cancer survivors than ever [8].

Treatment decision making is complex and besides potential benefits, side effects also have to be considered [9]. Lymphedema of hand, arm or shoulder are common side effects of breast cancer surgery and radiotherapy, affecting 10–50% of patients within the first three years after diagnosis [8, 10, 11]. Other long-term side effects related to local breast cancer therapy comprise numbness or tightness, and stretching or pulling in arms, shoulders, or the chest wall [7]. Both surgery and radiotherapy can lead to nerve damage resulting in chronic pain [11, 12] which may affect 25–60% of survivors after breast cancer treatment [8]. While short-term side effects of adjuvant chemotherapy predominantly occurring during the course of treatment have been studied extensively, there is still insufficient information on long-term side effects of several chemotherapeutic agents [13]. A common long-term side effect of chemotherapy is cardiotoxicity, as several cytotoxic agents, especially anthracyclines, can lead to cardiac complications [7, 8, 13]. Adjuvant systemic chemotherapy may also be associated with cognitive and neurological complications [7]. Peripheral neuropathy is a frequent side effect of treatment with chemotherapeutic agents, such as taxanes [14]. However, there is little evidence from long-term follow-up of patients with breast cancer receiving taxanes [11, 15].

Due to the growing number of breast cancer survivors treated with curative intent, more attention should be paid to the consequences of the often intensive treatment, especially in terms of the long-term symptoms which may negatively affect patients’ quality of life. In this work, we focus on the early (4 weeks and 6 months) and late (3 years) long-term symptoms of early breast cancer treatment after (neo)adjuvant chemotherapy in German routine practice. We present data from the MaTox project, a patient-reported outcomes survey within the clinical cohort study TMK (Tumour Registry Breast Cancer), which evaluated the frequency, severity, and persistence of typical symptoms in patients with breast cancer in routine practice using a questionnaire specifically designed for this purpose.

## Materials and methods

### Data source

The TMK is an ongoing, open, longitudinal, multicentre, observational, prospective cohort study which started in 2007. The study was reviewed by the responsible ethics committee and is registered at ClinicalTrials.gov (NCT01351584). Eligible patients are women aged ≥18 years with histologically confirmed breast cancer and systemic antineoplastic treatment. Written informed consent was obtained from all patients. A maximum of 6-week time difference was allowed between start of systemic therapy and signed informed consent. The TMK has previously been described in detail [16].

The MaTox project was a prospective, longitudinal survey within the TMK evaluating long-term impairments of treatment in patients with breast cancer receiving (neo)adjuvant systemic therapy. Between May 2008 and February 2009, 61 outpatient-centres for medical oncology located all over Germany actively recruited patients for the MaTox project. To minimize selection bias, study sites were encouraged to enrol patients consecutively and annual recruitment was restricted to six patients per study site per year. Patients filled in the MaTox questionnaire, which was specifically developed based on a review of the literature and an expert survey on potential long-term impairments after breast cancer treatment. Response categories were largely based on the severity assessments of the corresponding CTCAE (Common Terminology Criteria of Adverse Events), version 3.0 of the National Cancer Institute. A total of 26 items (symptoms) were scored on a 4-/5-point scale. The questionnaire was validated for clarity and comprehensibility in a small pilot study with patients filling in the questionnaire and giving feedback afterwards. If necessary, wording of the questions was revised. Patients received the first questionnaire via mail at enrolment and filled it out a median of 4.1 weeks after start of treatment (interquartile range 4.9 weeks). Further questionnaires were sent 6, 18 and 36 months (3 years) later. Reminders were sent via mail two weeks after distribution of the questionnaires and a second and last reminder another two weeks later. Delivery of questionnaires was ended if patients experienced a recurrence. This analysis focuses on early impairments 4 weeks and 6 months as well as on late impairments 3 years after start of systemic treatment. Symptoms reported at 18 months and 3 years differed only slightly in scoring and in the number of patients affected, and therefore only results of the 3 years time point are shown. Data from all time points for all items are compiled in Table S1. The response rate of the questionnaires was 94% after 4 weeks, 92% after 6 months and 83% after 3 years.

### Cohort definition

**Fig. 1:**
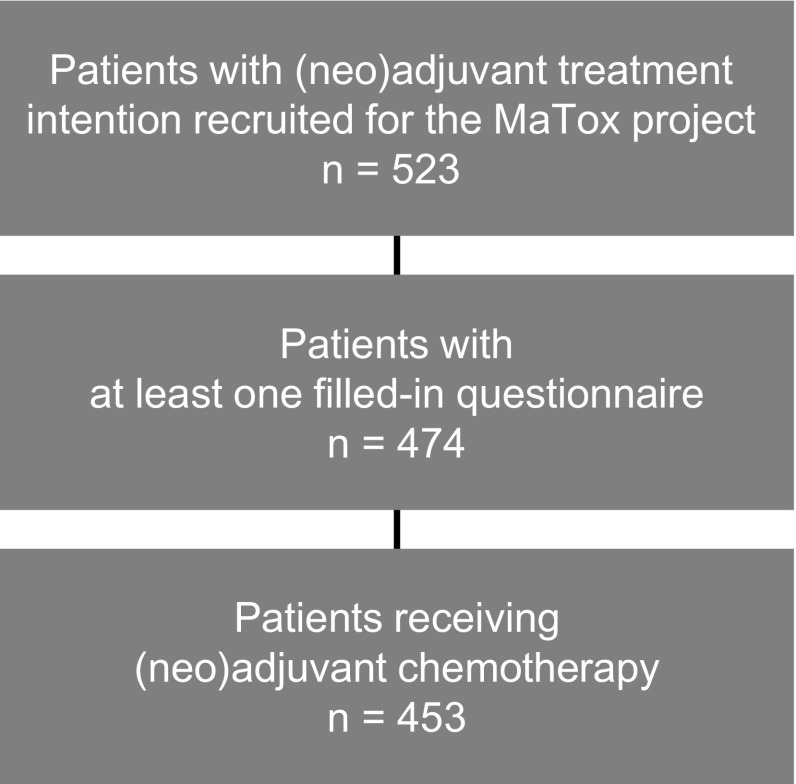
Cohort definition. Number of patients recruited for the MaTox project. All patients with at least one filled-in questionnaire receiving (neo)adjuvant chemotherapy were included into this analysis

523 patients with (neo)adjuvant treatment intention were recruited for the MaTox project between 2008 and 2009 (Fig. 1). Of these patients, 49 were excluded as minimum data on sociodemographics or treatment had been incomplete or not a single filled-in questionnaire had been sent back. Only patients who had received (neo)adjuvant chemotherapy were included in the present analysis, because the subgroup of patients who had received systemic endocrine treatment only was too small for a meaningful analysis. 3% of the patients had experienced recurrence or died after 6 months (13% of the patients after 3 years).

### Statistical analysis

A multivariate logistic regression model was used to identify potentially predictive factors for polyneuropathy (paraesthesia: ‘do you suffer from tingling or pain in fingers or toes’) and surgery (‘do you have pain in the arm/shoulder/chest wall at the operated site’) 3 years after start of chemotherapy (no symptoms vs. any severity of symptoms). The following independent variables were examined for the model on paraesthesia: age at primary diagnosis, Charlson Comorbidity Index [CCI, 17], diabetes, paraesthesia shortly after start of chemotherapy (median 4 weeks), treatment with taxane-based regimen and treatment with endocrine therapy. For the regression analysis on post-surgical symptoms, the variables included were age at primary diagnosis, Charlson Comorbidity Index [CCI, 17], tumour size (T stage), number of removed lymph nodes, resection of primary tumour by breast-conserving surgery, radiotherapy, pain in the arm/shoulder/chest wall at the operated side shortly after start of chemotherapy and treatment with endocrine therapy. Patients with missing parameters were excluded from the model. P-values are reported in an exploratory manner without adjustments for multiplicity. All analyses were performed using STATISTICA (StatSoft, Inc.) version 10.0 and R version 2.15.1.

## Results

### Patient, tumour and treatment characteristics

Table 1 presents the patient, tumour and treatment characteristics of the 453 patients included into this analysis. Median age at primary diagnosis was 57 years. 57% of the patients were postmenopausal at enrolment. 52% of the patients had hormone receptor (HR)-positive and human epidermal growth factor receptor 2 (HER2)-negative breast cancer. At least one comorbidity was present in 56% of patients, with hypertension mentioned most frequently (29%), followed by diabetes (8%). Overall, the burden by most serious comorbidities at diagnosis was low (78% CCI = 0).

**Table 1:** Patient and tumour characteristics

Characteristic	Patients (*n* = 453)	
	Median	Min–Max
Age at diagnosis (years)	57	30–79
	Mean	Std
BMI at enrolment	26.5	5.1
Patients with comorbidity^a^	*n*	%
Any comorbidity^b^	254	56.1%
CCI =0^c^	354	78.1%
CCI ≥1^c^	99	21.9%
Hypertension	130	28.7%
Diabetes	36	7.9%
Menopausal status^d^		
Premenopausal	127	28.0%
Perimenopausal	18	4.0%
Postmenopausal	260	57.4%
Unknown	48	10.6%
Receptor status^a^		
HR-positive, HER2-negative	237	52.3%
HR-positive, HER2-positive	76	16.8%
HR-negative, HER2-positive	38	8.4%
Triple negative	86	19.0%
Unknown	16	3.5%
Tumour stage^a,e^		
I	118	26.0%
II	201	44.4%
III	71	15.7%
Not determined/unknown^f^	63	13.9%
Nodal stage^a^		
Positive	222	49.0%
Negative (N0)	217	47.9%
Unknown (NX + missing)	14	3.1%
Location of primary tumour^a^		
Right	232	51.2%
Left	211	46.6%
Both	10	2.2%

*BMI* body mass index in kg/m^2^, *HR* hormone receptor, *HER2* human epidermal growth factor receptor 2, *Max* maximum, *Min* minimum, *N* regional lymph node, *Std* standard deviation

^a^At diagnosis

^b^Comorbidity according to Charlson [17] or additional concomitant diseases

^c^Charlson Comorbidity Index (CCI) according to Quan [18]

^d^At enrolment

^e^Tumour stage according to AJCC/UICC 7th edition

^f^For some patients, the exact stage could not be determined because of unknown parameters (TX, NX, MX)

Table 2 presents the treatment characteristics for the MaTox cohort. 66% of the patients underwent a breast-conserving resection of the primary tumour and 78% of all patients received radiotherapy. All patients included in this analysis underwent systemic chemotherapy, with 13% treated in the neoadjuvant and 87% in the adjuvant setting at enrolment. The majority of patients received an anthracycline-based regimen with epirubicin or doxorubicin (E/A) in combination with cyclophosphamide (C) and an additional substance. 86% of the patients with HER2-positive breast cancer were treated with trastuzumab plus chemotherapy. Approximately 61% of the patients received a taxane-based chemotherapy. Patients receiving a taxane in combination with E/A+C more frequently had positive lymph node status. In addition to chemotherapy, endocrine therapy was documented for 62% of all patients, corresponding to 90% of the patients with HR-positive tumours.

**Table 2:** Treatment characteristics

Treatment	Patients (*n* = 453)	
	*N*	%
Resection of primary tumour		
Breast-conserving (incl. follow-up resection)	299	66.0
Non-breast conserving (mastectomy/ablatio mammae)	130	28.7
Unknown	24	5.3
Radiotherapy received	354	78.1
Chemotherapy setting at enrolment^a^		
Neoadjuvant	58	12.8
Adjuvant	395	87.2
Taxane-based chemotherapy	275	60.7
Top chemotherapy regimen^b^		
F + E/A + C±Tra	151	33.3
F + E/A + C+D ± Tra	102	22.5
E/A + C+D ± Tra	61	13.5
E/A + C+P ± Tra	49	10.8
Car + D±Tra	18	4.0
Other	72	15.1
Endocrine therapy received	285	62.9
Endocrine therapy		
Aromatase inhibitors (AI) ± GnRH	120	26.5
Anti-oestrogen (AE)	96	21.2
Switch AI/AE	67	14.8
GnRH-analogue	2	0.4

*AE* anti-oestrogen, *AI* aromatase inhibitor, *C* cyclophosphamide, *Car* carboplatin, *D* docetaxel, *E/A* epirubicin/doxorubicin, *F* fluorouracil, *GnRH* gonadotropin-releasing hormone, *P* paclitaxel, *Tra* trastuzumab

^a^Only patients who had received (neo)adjuvant chemotherapy were included in the present analysis, because the subgroup of patients who had received endocrine treatment only was too small for a meaningful analysis

^b^At primary diagnosis

### Early and late long-term impairments

**Fig. 2:**
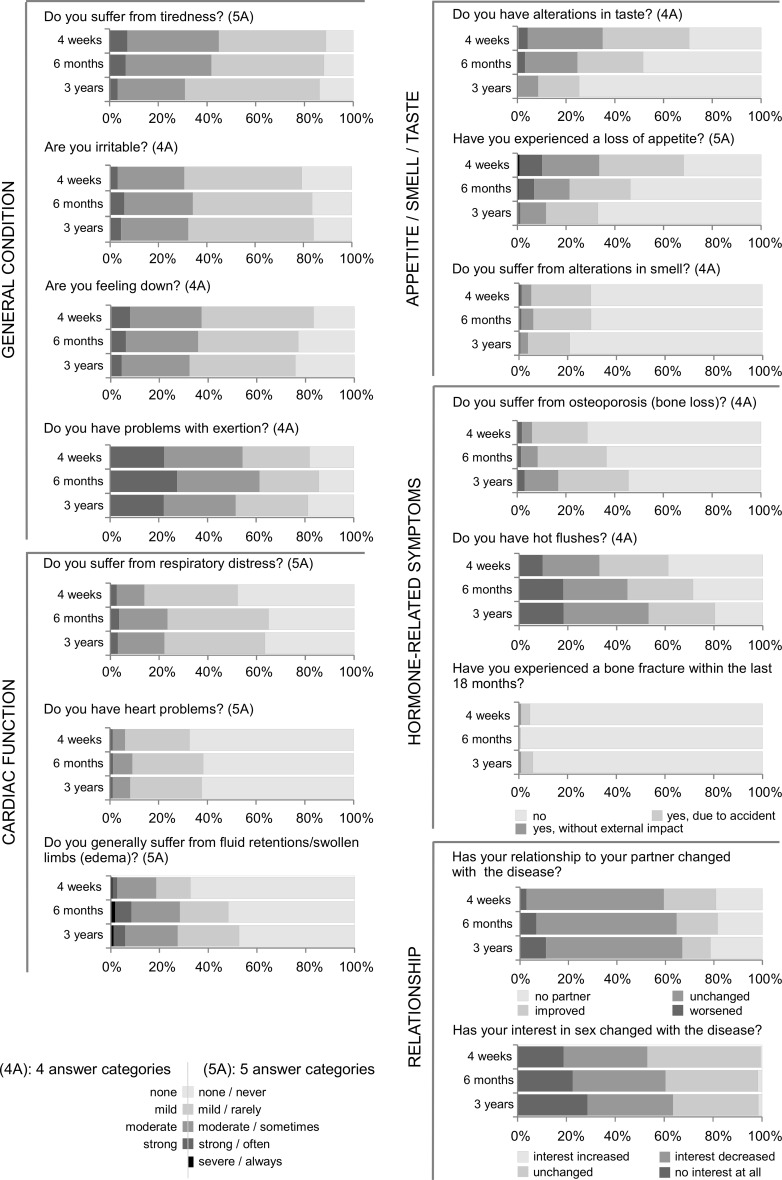
Results of the patient-reported outcomes—part I. Shown are the frequencies of severity of the reported symptoms 4 weeks, 6 months and 3 years after start of chemotherapy. *4A* four answer categories, *5A* five answer categories

Looking at the various symptoms reported over time, alterations in taste, smell, loss of appetite and fatigue improved over time: of 70% patients affected by symptoms of any severity in taste and 68% in loss of appetite 4 weeks after start of chemotherapy, only 26 and 33% reported long-term impairments, respectively (Fig. 2). 31% of the patients reported at least moderate fatigue 3 years after start of chemotherapy in contrast to more than 45% after 4 weeks. In contrast, hormone-related symptoms, such as osteoporosis and hot flushes, increased over time, especially in patients with endocrine therapy (data on file). There was also an increase in patients with moderate respiratory distress (14% after 4 weeks, 22% after 3 years), and a 20% increase in patients reporting problems with fluid retention/oedema.

**Fig. 3:**
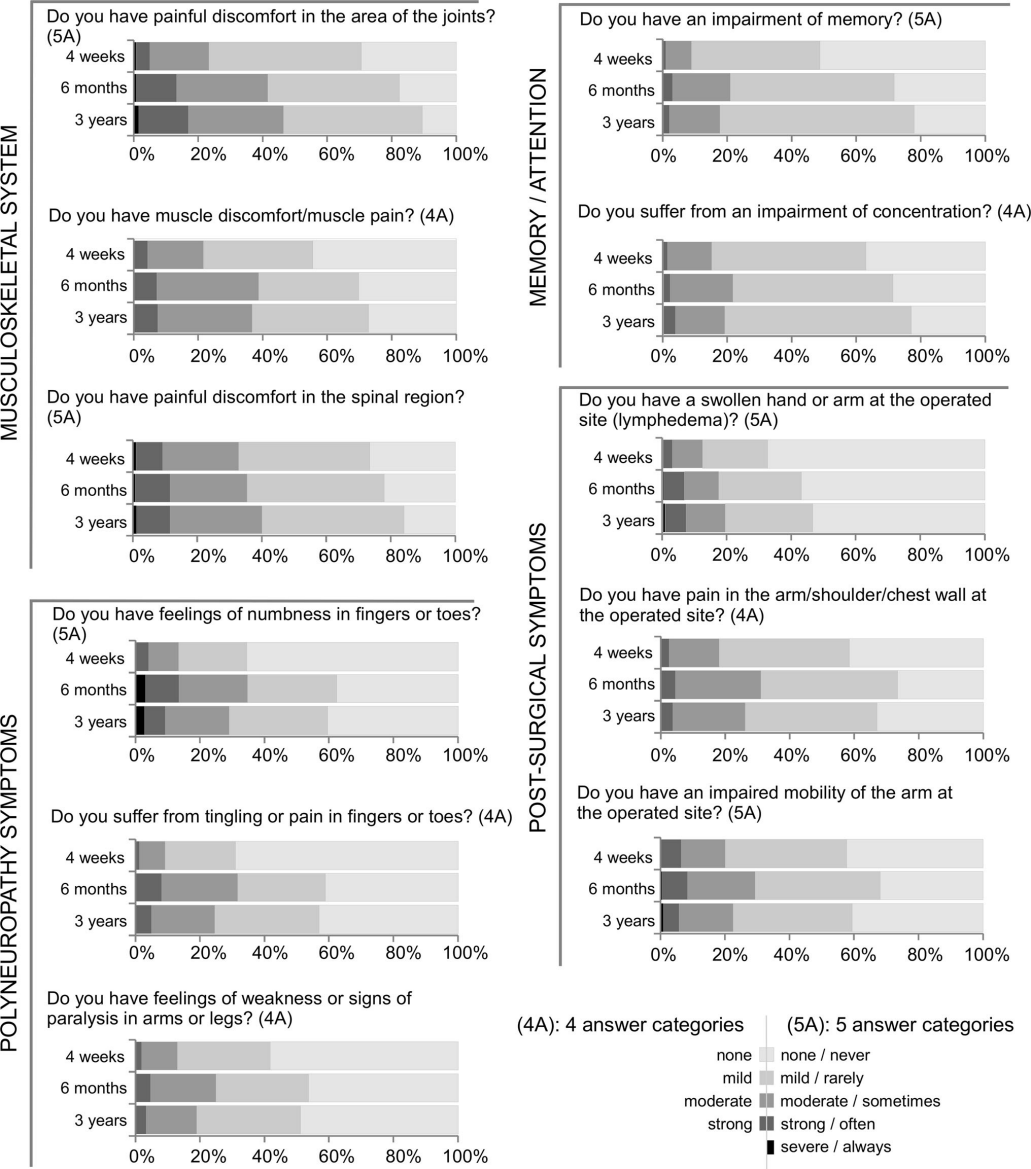
Results of the patient-reported outcomes—part II. Shown are the frequencies of severity of the reported symptoms 4 weeks, 6 months and 3 years after start of chemotherapy. *4A* four answer categories, *5A* five answer categories

Symptoms which increased most at 6 months and were persistent at 3 years related to the areas memory/attention, musculoskeletal system, post-surgical and polyneuropathy symptoms (Fig. 3). Memory/attention- and musculoskeletal system-associated symptoms increased over time, with most patients affected 3 years after treatment. Already 4 weeks after start of treatment, 56% of patients suffered from muscle discomfort/pain, increasing to 73% 3 years later. A small number of patients (5–8%) reported strong discomfort/pain at each time point. Patients with post-surgical symptoms still reported strong impairments at 3 years, especially in terms of lymphedema and impaired mobility of the arm at the operated site. 40% of the patients had mild and 19% moderate to strong pain in arm/shoulder/chest wall on the operated site 4 weeks after start of treatment. 3 years later, 40% reported mild pain, whereas 26% suffered from moderate to strong post-surgical pain (Fig. 3). Regarding polyneuropathy symptoms, 31% of the patients reported any severity of tingling or pain in fingers or toes (paraesthesia) already 4 weeks after start of chemotherapy. Both 6 months and 3 years later, their number increased to 60%, and up to 10% reported strong symptoms.

117 patients did not fill in the 3-year questionnaire: 14 patients (3%) had died, 47 patients (10%) had had a recurrence, 19 patients (4%) had been lost to follow-up and 37 patients (8%) had not sent back the questionnaire for unknown reasons. In order to check whether the patients who had not sent back the 3-year questionnaire differed at the start of treatment from those who had sent it back, we compared the patient and treatment characteristics at enrolment as well as the PROs of the first questionnaire (Tables S2 and S3). The patients who had not sent back the 3-year questionnaire were slightly younger (median age 54 vs. 57 years), more often had comorbidities (31 vs. 19% CCI ≥1), triple-negative receptor status (27 vs. 16%), neoadjuvant chemotherapy (25 vs. 9%) and a non-breast-conserving resection of the tumour (36 vs. 26%, Table S2). There were no striking differences between the PROs of the two subgroups; those patients who had not sent back the 3-year questionnaire reported slightly more often irritability, problems with exertion, memory, attention and strong muscle pain, yet less often tiredness, hot flushes and pain in the spinal region (Table S3).

Baseline data before treatment are generally not available in the oncological setting. In our survey, the first questionnaire was filled in after surgery, at the start of systemic therapy. To investigate a potential effect of the timing of the first questionnaire, we analysed all items of the first questionnaire according to the fill-in date. The diagrams are depicted in Figure S1. No major differences were seen in most items between patients who filled in the first questionnaire during the first cycle and those who filled it in at later time points. PROs on the items “pain at the operated site” and “impaired mobility of the arm at the operated site” improved substantially the more time had passed since surgery. Regarding the paraesthesia symptoms, there was no difference according to the fill-in date. The PROs on the items “alterations in taste” and “impaired memory” worsened markedly during the first weeks of systemic treatment (Figure S1).

### Risk factors for developing post-surgical and paraesthesia symptoms

We took a closer look at paraesthesia and post-surgery pain using multivariate logistic regression models (Table 3). The results show that patients with paraesthesia symptoms 4 weeks after start of chemotherapy had an almost four-fold increased risk of paraesthesia of any severity 3 years later (OR 3.72, 95% CI 2.15–6.67, *p* < 0.0001). Likewise, older age at diagnosis or receiving a taxane-based chemotherapy was associated with an increased risk of long-term paraesthesia (age, +10 years: OR 1.34, 95% CI 1.07–1.68, *p* < 0.05; taxane-based regimen: OR 1.65, 95% CI 1.01–2.69, *p* < 0.05). Diabetes was not found to be an independent risk factor for paraesthesia 3 years after start of chemotherapy in the multivariate model adjusted for paraesthesia at start of chemotherapy (amongst others). Regarding the post-surgical symptoms, patients reporting pain of any severity 4 weeks after start of chemotherapy, e.g. after surgery and before radiotherapy, had a more than four-fold risk of still having those symptoms 3 years later (OR 4.52, 95% CI 2.62–7.95, *p* < 0.0001). A tendency towards more pain 3 years after treatment was associated with radiotherapy of the breast region (OR 2.10, 95% CI 0.96–4.65, *p* = 0.0648). There was no significant correlation between postoperative symptoms and age, CCI, tumour size, number of removed lymph nodes, breast-conserving surgery, radiotherapy or endocrine therapy (Table 3).

**Table 3:** Multivariate logistic regression analysis

	OR (95% CI)	*p* value
Paraesthesia 3 years after start of CTx (*n* = 312^a^)		
**Age (years, +10)** ^**b**^	**1.34 (1.07–1.68)**	**0.0105**
CCI (+1)	1.06 (0.72–1.61)	0.7589
Diabetes (yes vs. no)	1.71 (0.50–6.60)	0.4089
**Paraesthesia 4** **weeks after start of CTx (yes vs. no)**	**3.72 (2.15–6.67)**	**<0.0001**
**Taxane-based regimen (yes vs. no)**	**1.65 (1.01–2.69)**	**0.0447**
Endocrine therapy(yes vs. no)	1.21 (0.73–2.01)	0.4639
Post-surgical symptoms 3 years after start of CTx (*n* = 269^c^)		
Age (years, +10)^b^	0.89 (0.69–1.14)	0.3692
CCI (+1)	1.01 (0.70-1.52)	0.9421
Tumour size(Tis,T1,T2 vs. T3,T4,TX)	2.43 (0.72–8.55)	0.1544
Number of removed lymph nodes (+1)	1.01 (0.98–1.04)	0.6153
Breast-conserving surgery (yes vs. no)^d^	0.76 (0.34–1.65)	0.4874
Radiotherapy (lymph node/thorax vs. none)	2.00 (0.75–5.53)	0.1709
Radiotherapy (mamma vs. none)	2.10 (0.96–4.65)	0.0648
**Pain at the operated site 4** **weeks after start of CTx (yes vs. no)**	**4.52 (2.62–7.95)**	**<0.0001**
Endocrine therapy(yes vs. no)	0.66 (0.35–1.21)	0.1883

*Bold writing p* < 0.05

*CCI* Charlson comorbidity index, *CI* confidence interval, *CTx* chemotherapy, *OR* odds ratio

^a^Of 453 patients, 141 were excluded: 19 because of at least one parameter missing and 122 had not sent back the 3-year questionnaire; 176 of 312 patients reported symptoms of paraesthesia 3 years after start of chemotherapy

^b^At diagnosis

^c^Of 453 patients, 184 were excluded: 62 because of at least one parameter missing and 122 had not sent back the 3-year questionnaire; 178 of 269 patients reported post-surgical symptoms 3 years after start of chemotherapy

^d^Of the primary tumour

## Discussion

The MaTox project assessed long-term well-being after early breast cancer treatment in 453 patients receiving surgery, chemotherapy, and some also radiation and/or endocrine therapy in German routine practice. Our data show that especially impairments in memory/attention and symptoms of the musculoskeletal system as well as symptoms associated with surgery and neuropathy increased over time and were persistent 3 years after start of systemic treatment. Patients had a substantially increased risk of paraesthesia if those symptoms appeared shortly after start of treatment or if taxanes were given.

One limitation of this project is the exclusive enrolment of patients receiving chemotherapy, limiting generalizability regarding patients that only received endocrine treatment. Not all initially participating patients sent back the subsequent questionnaires, representing a potential limitation. Data comparing patients’ tumour and treatment characteristics as well as PROs at enrolment between patients who had sent back the 3-year questionnaire and those who had not show that patients in impaired condition (comorbidities at start of treatment), with inferior prognosis (triple-negative tumour, non-breast-conserving surgery) or with neoadjuvant chemotherapy treatment, are potentially slightly under-represented at 3 years. However, the return rate of the questionnaires is exceptionally high (>83% of patients alive and without recurrence at each time point), strengthening the generalizability of our data. Strengths of this project are the prospective, longitudinal data collection and the participation of oncologists from all over Germany recruiting a large, representative study cohort.

As soon as a woman is diagnosed with breast cancer, her quality of life is affected. Shock, sadness, anxiety, fatigue and depression are only some of the psychologically distressing symptoms which lead to a decreased quality of life [18, 19]. Longitudinal assessment including pre-diagnosis data is hardly feasible in the oncological setting, and capturing data prior to systemic therapy is hardly possible in the non-interventional setting. Thus, the focus of our study was to assess which symptoms are present long after the initial treatment with surgery, systemic therapy and radiation independent of causal relation. We show the patients’ symptom burden at early and late time points after start of systemic treatment. It was striking to see that 3 years after start of chemotherapy, considerable symptoms persisted and affected patient’s well-being. While deterioration of symptoms associated with memory/attention and the musculoskeletal system could be due to factors not related to treatment (e.g. ageing), increased postoperative pain and paraesthesia are likely a result of the curative treatment approach.

The proportion of patients affected by an impairment of memory in our cohort increased from 49% at start to 78% after 3 years. Cognitive impairment as a late effect of cancer treatment has been studied for several years [6, 11] and may comprise problems with memory/attention, learning, speed in mental processing, and executive or sensorimotor functions [8]. Cognitive deficits have been associated with adjuvant chemotherapy [6, 9]. However, several studies showing this association have been criticized due to methodological aspects such as heterogeneity in the types of chemotherapy applied, differences in assessing cognitive function and varieties in study duration [13]. Furthermore, studies often did not control for potential confounders such as mood, menopausal status, endocrine therapy [13] or demential syndromes, especially in older women [6]. It is known that chemotherapy-related fatigue, depression and anxiety can contribute to poor cognitive performance [7]. Thus, cognitive impairment seems to be linked to several factors besides systemic treatment. Independent of its cause, we believe the high frequency of this impairment reported in our project highlights a need for better management of cognitive dysfunction in cancer survivors, as also claimed by others [8].

Cardiotoxicity is a well-known side effect of cytotoxic therapy [20]. While reports about heart problems were low after 3 years in our cohort, there was a slight increase in patients reporting mild to strong respiratory symptoms or fluid retentions/oedema. These symptoms might be due to various reasons but could also be a sign for development of heart problems. As this long-term toxicity influences patients’ quality of life, efforts for prevention, treatment and follow-up care for cardiotoxicity are strongly encouraged [7, 8, 20].

The development of musculoskeletal symptoms is a multicausal process, with endocrine therapy increasing the risk [21]. According to our data, symptoms of the musculoskeletal system were present in at least half of the patients 4 weeks after start of treatment, but had further increased after 3 years, especially in patients who received endocrine therapy, confirming previous observations [22]. However, it should be kept in mind that age is also associated with an increasing incidence of musculoskeletal symptoms [23, 24].

Postoperative impairments comprise lymphedema, pain and mobility at the operated site. Lymphedema and subsequent swelling in the arm or chest is caused by insufficient lymph transport due to lymph node dissection and radiotherapy [8, 25]. 46% of the MaTox patients reported minor to severe lymphedema 3 years after start of treatment. This number is consistent with previous studies, reporting that 10–50% of patients with breast cancer are affected by lymphedema [7, 11]. Persistent pain after surgical treatment is also a well-known clinical problem in 25–50% of patients. Gärtner and colleagues showed that 47% of breast cancer patients still reported lingering pain years after surgery [26]. While 73% of the MaTox patients (42% mild, 26% moderate and 5% strong) suffered from pain at the operated site 6 months after start of treatment, this number decreased only slightly to 67% (41% mild, 22% moderate and 4% strong) 3 years later. In our cohort, pain after surgery, which was persistent at 4 weeks after start of chemotherapy, was identified as a risk factor for long-term pain after breast cancer surgery. This is in accordance with other studies [26, 27], whereas the risk factors age and tumour size could not be confirmed in our cohort [8]. Although nerve-sparing operation techniques have reduced the incidence of chronic pain, additional strategies for improvement of the patients’ situation are obviously needed.

In the MaTox cohort, 30% of the patients reported symptoms of paraesthesia as early as 4 weeks after start of treatment and almost 60% of the patients reported paraesthesia symptoms 3 years later. Paraesthesia is often an early sign of chemotherapy-induced neuropathy, which may greatly affect patients’ quality of life [14, 28, 29]. The extent of neuropathy is dependent on the chemotherapy agent [14, 30–32], the dose used and may persist for years [7, 33]. Logistic regression analysis in the MaTox cohort showed that patients with higher age and early paraesthesia symptoms as well as those patients receiving taxane-based chemotherapy had a higher risk of suffering from paraesthesia 3 years after treatment. It would be interesting to investigate whether early intervention in these patients, e.g. dose adjustment, could reduce the frequency of long-term neuropathy.

## Conclusion

The MaTox project collected patient-reported outcomes after (neo)adjuvant systemic breast cancer treatment and identified long-term impairments. Even 3 years after start of treatment, more than half of the patients had paraesthesia symptoms and about two-thirds of the patients still reported pain at the operated site. It is important to discuss these long-term toxicities with patients, and to identify treatments and management strategies that decrease the impact of these impairments on the quality of life of breast cancer survivors.

## Electronic supplementary material

Below is the link to the electronic supplementary material.

Supplementary material 1 (PDF 254 kb)

Supplementary material 2 (PDF 267 kb)

Supplementary material 3 (PDF 166 kb)

Supplementary material 4 (PDF 176 kb)
