# Supplementary figures and images for: Persistent impairments 3 years after (neo)adjuvant chemotherapy for breast cancer: results from the MaTox project
Source: Breast Cancer Res Treat. 2017 Jul 5;165(3):721–31. doi: 10.1007/s10549-017-4365-7 (PMC5602000; doi:10.1007/s10549-017-4365-7)

Figure S1 - PRO for the first questionnaire according to the fill-in date

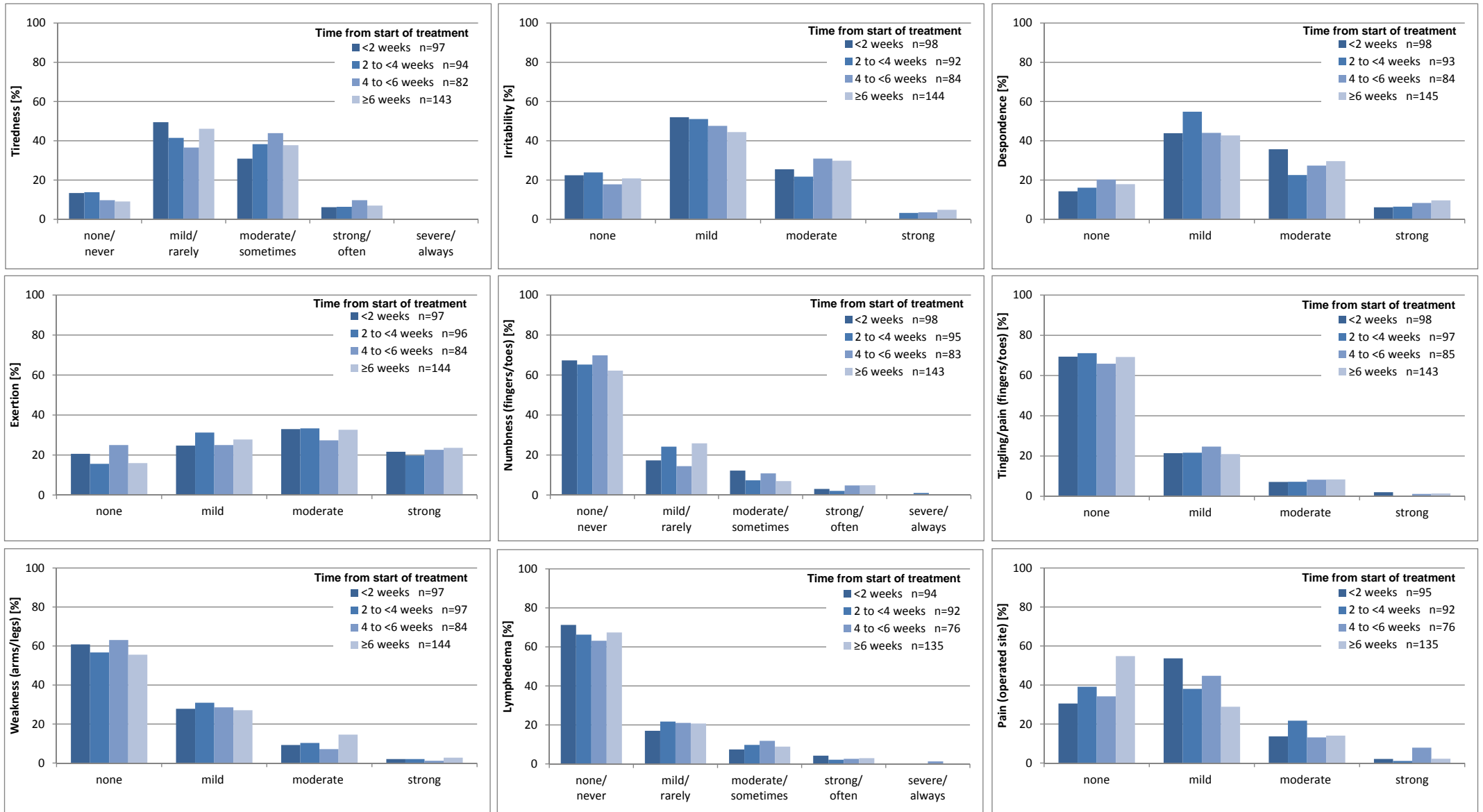

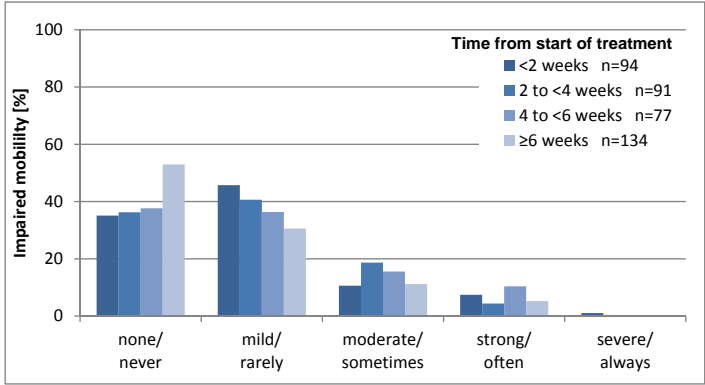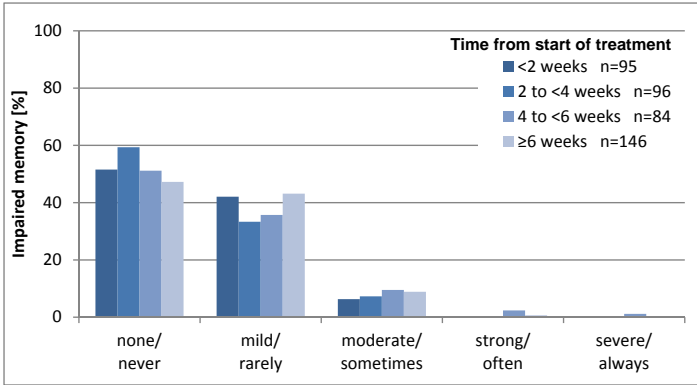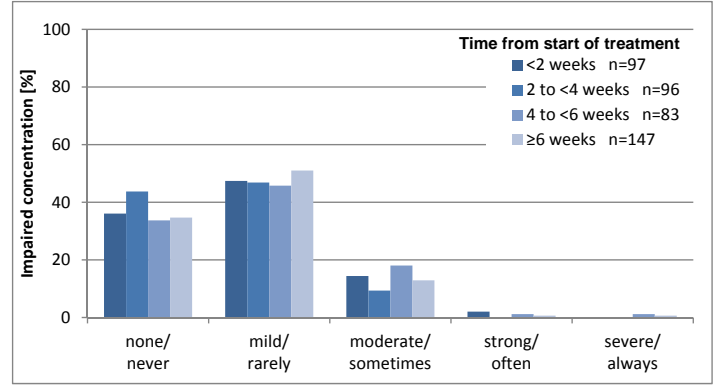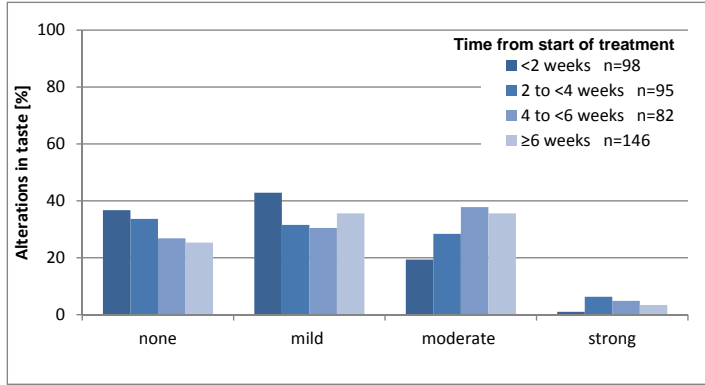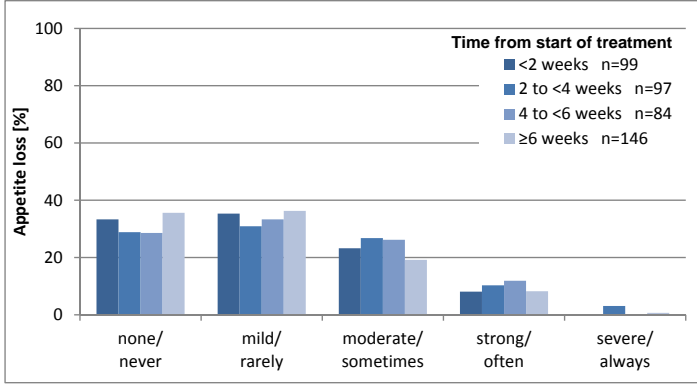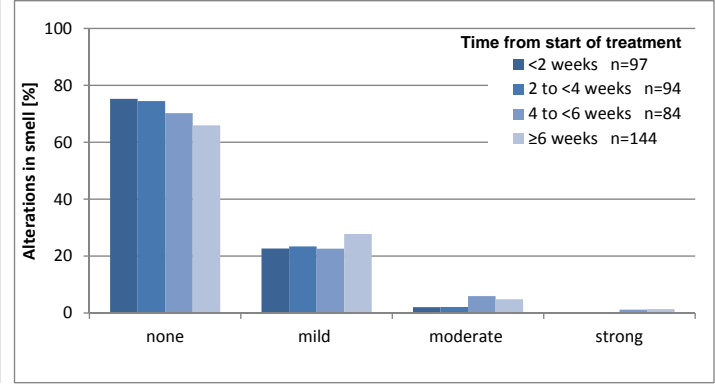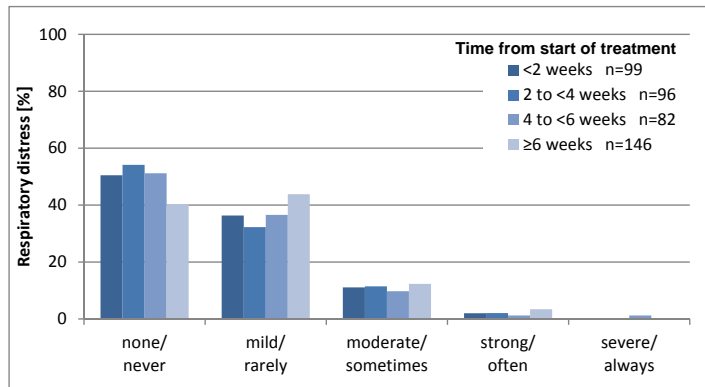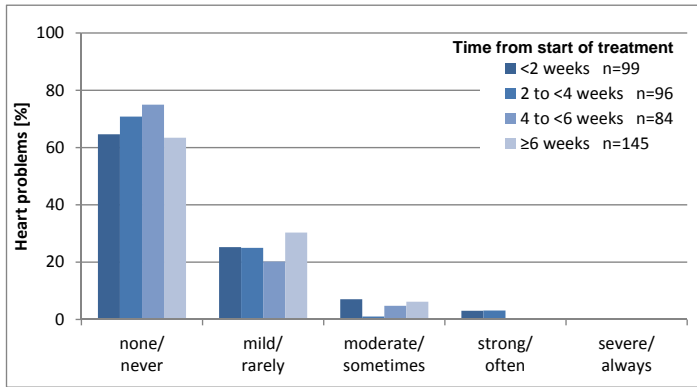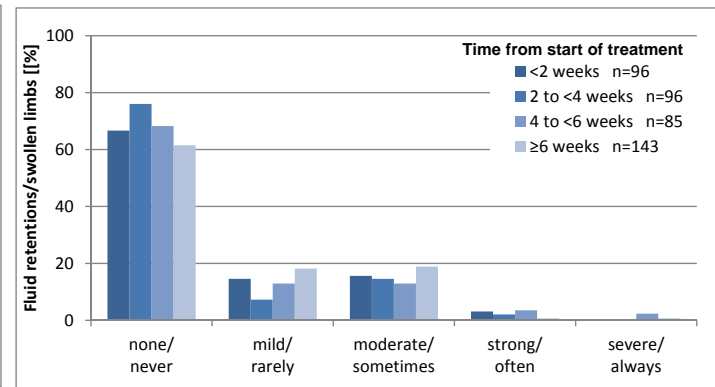

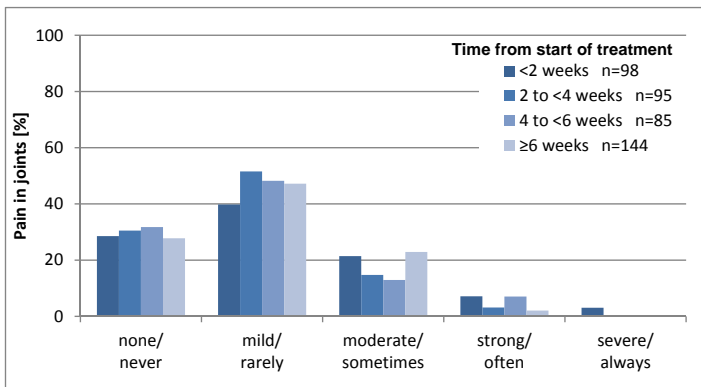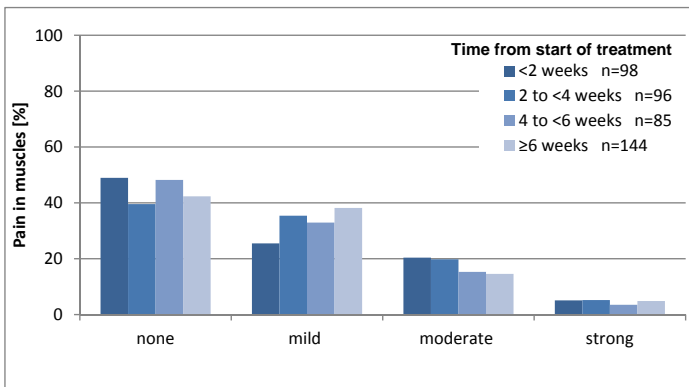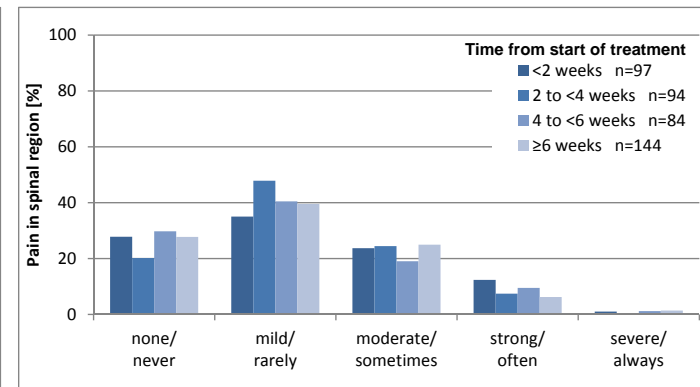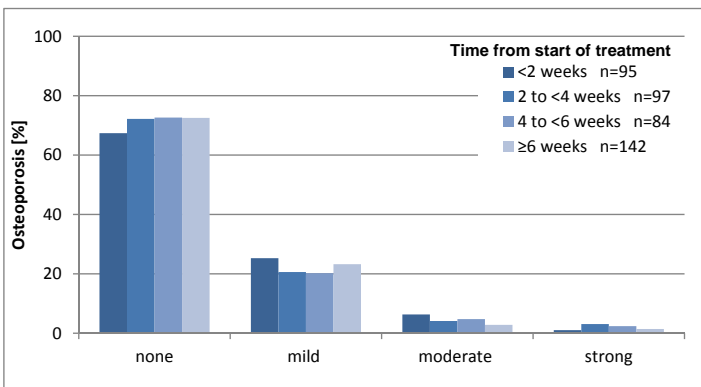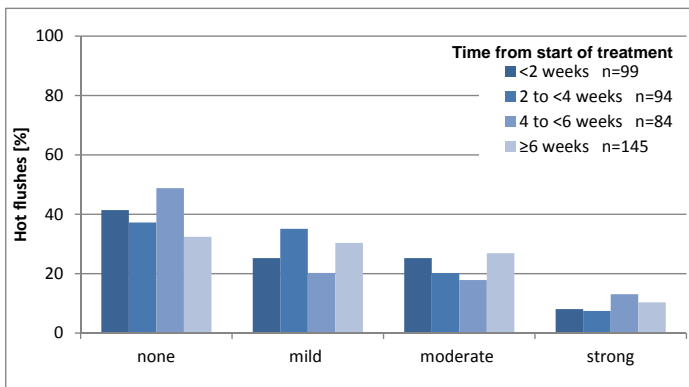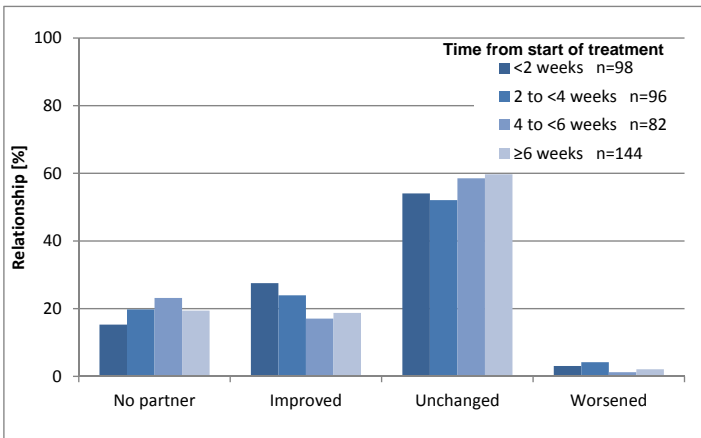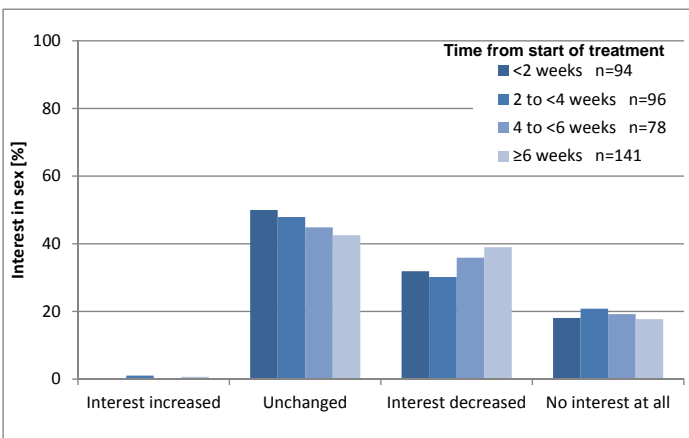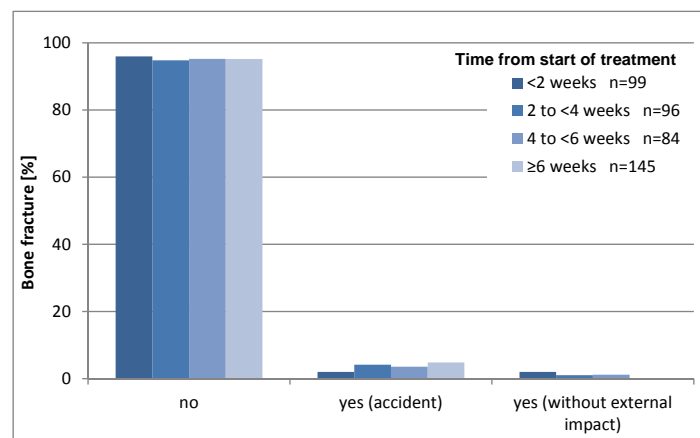

Supplement: Supplementary file 1 — Supplementary material 1 (PDF 254 kb) [file 10549_2017_4365_MOESM1_ESM.pdf]
