# Supplementary material for: Persistent impairments 3 years after (neo)adjuvant chemotherapy for breast cancer: results from the MaTox project
Source: Breast Cancer Res Treat. 2017 Jul 5;165(3):721–31. doi: 10.1007/s10549-017-4365-7 (PMC5602000; doi:10.1007/s10549-017-4365-7)
Supplement: Supplementary file 2 — Supplementary material 2 (PDF 267 kb) [file 10549_2017_4365_MOESM2_ESM.pdf]

## Table S1. Results of the patient reported outcomes – all timepoints

| Item | 4 weeks | | 6 months | | 18 months | | 3 years | |
|---|---|---|---|---|---|---|---|---|
| | n | % | n | % | n | % | n | % |
| <u>**General condition**</u> | | | | | | | | |
| **Tiredness** | 416 | - | 398 | - | 376 | - | 332 | - |
| None / never | 47 | 11.3 | 50 | 12.6 | 63 | 16.8 | 47 | 14.2 |
| Mild / rarely | 183 | 44.0 | 182 | 45.7 | 192 | 51.1 | 182 | 54.8 |
| Moderate / sometimes | 156 | 37.5 | 139 | 34.9 | 111 | 29.5 | 92 | 27.7 |
| Strong / often | 30 | 7.2 | 27 | 6.8 | 10 | 2.7 | 10 | 3.0 |
| Severe / always | 0 | - | 0 | - | 0 | - | 1 | 0.3 |
| **Irritability** | 418 | - | 401 | - | 376 | - | 334 | - |
| None | 89 | 21.3 | 68 | 17.0 | 65 | 17.3 | 53 | 15.9 |
| Mild | 202 | 48.3 | 196 | 48.9 | 189 | 50.3 | 174 | 52.1 |
| Moderate | 114 | 27.3 | 114 | 28.4 | 107 | 28.5 | 92 | 27.5 |
| Strong | 13 | 3.1 | 23 | 5.7 | 15 | 4.0 | 15 | 4.5 |
| **Despondence** | 420 | - | 402 | - | 375 | - | 333 | - |
| None | 72 | 17.1 | 93 | 23.1 | 85 | 22.7 | 81 | 24.3 |
| Mild | 193 | 46.0 | 164 | 40.8 | 159 | 42.4 | 145 | 43.5 |
| Moderate | 122 | 29.0 | 120 | 29.9 | 117 | 31.2 | 92 | 27.6 |
| Strong | 33 | 7.9 | 25 | 6.2 | 14 | 3.7 | 15 | 4.5 |
| **Exertion** | 421 | - | 399 | - | 377 | - | 332 | - |
| None | 79 | 18.8 | 56 | 14.0 | 85 | 22.5 | 62 | 18.7 |
| Mild | 115 | 27.3 | 98 | 24.6 | 104 | 27.6 | 99 | 29.8 |
| Moderate | 134 | 31.8 | 136 | 34.1 | 120 | 31.8 | 98 | 29.5 |
| Strong | 93 | 22.1 | 109 | 27.3 | 68 | 18.0 | 73 | 22.0 |
| <u>**Polyneuropathy symptoms**</u> | | | | | | | | |
| **Numbness (fingers/toes)** | 419 | - | 397 | - | 377 | - | 330 | - |
| None / never | 275 | 65.6 | 147 | 37.0 | 168 | 44.6 | 133 | 40.3 |
| Mild / rarely | 89 | 21.2 | 111 | 28.0 | 88 | 23.3 | 101 | 30.6 |
| Moderate / sometimes | 38 | 9.1 | 85 | 21.4 | 80 | 21.2 | 66 | 20.0 |
| Strong / often | 16 | 3.8 | 42 | 10.6 | 35 | 9.3 | 21 | 6.4 |
| Severe / always | 1 | 0.2 | 12 | 3.0 | 6 | 1.6 | 9 | 2.7 |
| **Tingling / pain (fingers/toes)** | 423 | - | 397 | - | 371 | - | 331 | - |
| None | 292 | 69.0 | 161 | 40.6 | 162 | 43.7 | 142 | 42.9 |
| Mild | 93 | 22.0 | 109 | 27.5 | 99 | 26.7 | 108 | 32.6 |
| Moderate | 33 | 7.8 | 94 | 23.7 | 94 | 25.3 | 65 | 19.6 |
| Strong | 5 | 1.2 | 33 | 8.3 | 16 | 4.3 | 16 | 4.8 |
| **Weakness (arms/legs)** | 422 | - | 395 | - | 376 | - | 332 | - |
| None | 247 | 58.5 | 183 | 46.3 | 180 | 47.9 | 162 | 48.8 |
| Mild | 120 | 28.4 | 113 | 28.6 | 121 | 32.2 | 107 | 32.2 |
| Moderate | 46 | 10.9 | 81 | 20.5 | 60 | 16.0 | 52 | 15.7 |
| Strong | 9 | 2.1 | 18 | 4.6 | 15 | 4.0 | 11 | 3.3 |

| | | | | | | | | |
|---|---|---|---|---|---|---|---|---|
| **<u>Post-surgical symptoms</u>** | | | | | | | | |
| **Lymphedema** | 397 | - | 400 | - | 376 | - | 334 | - |
| None / never | 267 | 67.3 | 226 | 56.5 | 201 | 53.5 | 177 | 53.0 |
| Mild / rarely | 80 | 20.2 | 104 | 26.0 | 90 | 23.9 | 91 | 27.2 |
| Moderate / sometimes | 37 | 9.3 | 43 | 10.8 | 54 | 14.4 | 42 | 12.6 |
| Strong / often | 12 | 3.0 | 25 | 6.3 | 27 | 7.2 | 21 | 6.3 |
| Severe / always | 1 | 0.3 | 2 | 0.5 | 4 | 1.1 | 3 | 0.9 |
| **Pain (operated site)** | 398 | - | 397 | - | 379 | - | 331 | - |
| None | 165 | 41.5 | 106 | 26.7 | 110 | 29.0 | 110 | 33.2 |
| Mild | 159 | 40.0 | 168 | 42.3 | 150 | 39.6 | 134 | 40.5 |
| Moderate | 62 | 15.6 | 105 | 26.4 | 103 | 27.2 | 74 | 22.4 |
| Strong | 12 | 3.0 | 18 | 4.5 | 16 | 4.2 | 13 | 3.9 |
| **Impaired mobility** | 396 | - | 398 | - | 374 | - | 334 | - |
| None / never | 166 | 41.9 | 127 | 31.9 | 132 | 35.3 | 136 | 40.7 |
| Mild / rarely | 149 | 37.6 | 154 | 38.7 | 140 | 37.4 | 122 | 36.5 |
| Moderate / sometimes | 54 | 13.6 | 84 | 21.1 | 64 | 17.1 | 57 | 17.1 |
| Strong / often | 26 | 6.6 | 31 | 7.8 | 37 | 9.9 | 16 | 4.8 |
| Severe / always | 1 | 0.3 | 2 | 0.5 | 1 | 0.3 | 3 | 0.9 |
| **<u>Memory / attention</u>** | | | | | | | | |
| **Impaired memory** | 421 | - | 402 | - | 380 | - | 333 | - |
| None / never | 218 | 51.8 | 113 | 28.1 | 105 | 27.6 | 74 | 22.2 |
| Mild / rarely | 165 | 39.2 | 205 | 51.0 | 197 | 51.8 | 201 | 60.4 |
| Moderate / sometimes | 34 | 8.1 | 72 | 17.9 | 66 | 17.4 | 51 | 15.3 |
| Strong / often | 3 | 0.7 | 12 | 3.0 | 11 | 2.9 | 6 | 1.8 |
| Severe / always | 1 | 0.2 | 0 | - | 1 | 0.3 | 1 | 0.3 |
| **Impaired concentration** | 423 | - | 398 | - | 378 | - | 333 | - |
| None / never | 156 | 36.9 | 113 | 28.4 | 98 | 25.9 | 76 | 22.8 |
| Mild / rarely | 204 | 48.2 | 200 | 50.3 | 204 | 54.0 | 194 | 58.3 |
| Moderate / sometimes | 57 | 13.5 | 76 | 19.1 | 63 | 16.7 | 50 | 15.0 |
| Strong / often | 4 | 0.9 | 9 | 2.3 | 13 | 3.4 | 13 | 3.9 |
| Severe / always | 2 | 0.5 | 0 | - | 0 | - | 0 | - |
| **<u>Appetite / smell / taste</u>** | | | | | | | | |
| **Alterations in taste** | 421 | - | 383 | - | 366 | - | 333 | - |
| None | 127 | 30.2 | 185 | 48.3 | 252 | 68.9 | 248 | 74.5 |
| Mild | 149 | 35.4 | 103 | 26.9 | 73 | 19.9 | 56 | 16.8 |
| Moderate | 129 | 30.6 | 82 | 21.4 | 35 | 9.6 | 28 | 8.4 |
| Strong | 16 | 3.8 | 13 | 3.4 | 6 | 1.6 | 1 | 0.3 |
| **Appetite loss** | 426 | - | 399 | - | 375 | - | 330 | - |
| None / never | 137 | 32.2 | 215 | 53.9 | 256 | 68.3 | 221 | 67.0 |
| Mild / rarely | 146 | 34.3 | 98 | 24.6 | 78 | 20.8 | 70 | 21.2 |
| Moderate / sometimes | 99 | 23.2 | 57 | 14.3 | 30 | 8.0 | 35 | 10.6 |
| Strong / often | 40 | 9.4 | 27 | 6.8 | 10 | 2.7 | 3 | 0.9 |
| Severe / always | 4 | 0.9 | 2 | 0.5 | 1 | 0.3 | 1 | 0.3 |

| | | | | | | | | |
|---|---|---|---|---|---|---|---|---|
| **Alterations in smell** | 419 | - | 399 | - | 373 | - | 330 | - |
| None | 297 | 70.9 | 281 | 70.4 | 294 | 78.8 | 260 | 78.8 |
| Mild | 103 | 24.6 | 95 | 23.8 | 63 | 16.9 | 58 | 17.6 |
| Moderate | 16 | 3.8 | 19 | 4.8 | 10 | 2.7 | 10 | 3.0 |
| Strong | 3 | 0.7 | 4 | 1.0 | 6 | 1.6 | 2 | 0.6 |
| **Cardiac function** | | | | | | | | |
| **Respiratory distress** | 423 | - | 397 | - | 374 | - | 331 | - |
| None / never | 203 | 48.0 | 138 | 34.8 | 145 | 38.8 | 119 | 36.0 |
| Mild / rarely | 161 | 38.1 | 165 | 41.6 | 156 | 41.7 | 138 | 41.7 |
| Moderate / sometimes | 48 | 11.3 | 79 | 19.9 | 60 | 16.0 | 62 | 18.7 |
| Strong / often | 10 | 2.4 | 14 | 3.5 | 11 | 2.9 | 12 | 3.6 |
| Severe / always | 1 | 0.2 | 1 | 0.3 | 2 | 0.5 | 0 | - |
| **Heart problems** | 424 | - | 398 | - | 371 | - | 332 | - |
| None / never | 287 | 67.7 | 246 | 61.8 | 240 | 64.7 | 207 | 62.3 |
| Mild / rarely | 110 | 25.9 | 116 | 29.1 | 100 | 27.0 | 97 | 29.2 |
| Moderate / sometimes | 21 | 5.0 | 31 | 7.8 | 22 | 5.9 | 24 | 7.2 |
| Strong / often | 6 | 1.4 | 5 | 1.3 | 9 | 2.4 | 4 | 1.2 |
| Severe / always | 0 | - | 0 | - | 0 | - | 0 | - |
| **Fluid retentions/swollen limbs** | 420 | - | 398 | - | 372 | - | 326 | - |
| None / never | 283 | 67.4 | 208 | 52.3 | 184 | 49.5 | 154 | 47.2 |
| Mild / rarely | 58 | 13.8 | 78 | 19.6 | 78 | 21.0 | 82 | 25.2 |
| Moderate / sometimes | 67 | 16.0 | 79 | 19.8 | 80 | 21.5 | 70 | 21.5 |
| Strong / often | 9 | 2.1 | 26 | 6.5 | 26 | 7.0 | 16 | 4.9 |
| Severe / always | 3 | 0.7 | 7 | 1.8 | 4 | 1.1 | 4 | 1.2 |
| **Musculoskeletal system** | | | | | | | | |
| **Pain joints** | 422 | - | 398 | - | 373 | - | 330 | - |
| None / never | 124 | 29.4 | 70 | 17.6 | 46 | 12.3 | 35 | 10.6 |
| Mild / rarely | 197 | 46.7 | 163 | 41.0 | 159 | 42.6 | 142 | 43.0 |
| Moderate / sometimes | 79 | 18.7 | 112 | 28.1 | 106 | 28.4 | 97 | 29.4 |
| Strong / often | 19 | 4.5 | 50 | 12.6 | 50 | 13.4 | 51 | 15.5 |
| Severe / always | 3 | 0.7 | 3 | 0.8 | 12 | 3.2 | 5 | 1.5 |
| **Pain muscles** | 423 | - | 396 | - | 371 | - | 330 | - |
| None | 188 | 44.4 | 120 | 30.3 | 91 | 24.5 | 90 | 27.3 |
| Mild | 142 | 33.6 | 123 | 31.1 | 130 | 35.0 | 119 | 36.1 |
| Moderate | 73 | 17.3 | 124 | 31.3 | 122 | 32.9 | 96 | 29.1 |
| Strong | 20 | 4.7 | 29 | 7.3 | 28 | 7.5 | 25 | 7.6 |
| **Pain spinal region** | 419 | - | 396 | - | 376 | - | 333 | - |
| None / never | 111 | 26.5 | 87 | 22.0 | 71 | 18.9 | 54 | 16.2 |
| Mild / rarely | 170 | 40.6 | 169 | 42.7 | 173 | 46.0 | 145 | 43.5 |
| Moderate / sometimes | 98 | 23.4 | 93 | 23.5 | 84 | 22.3 | 95 | 28.5 |
| Strong / often | 36 | 8.6 | 43 | 10.9 | 42 | 11.2 | 35 | 10.5 |
| Severe / always | 4 | 1.0 | 4 | 1.0 | 6 | 1.6 | 4 | 1.2 |

| | | | | | | | | |
|---|---|---|---|---|---|---|---|---|
| **Hormone-related symptoms** | | | | | | | | |
| **Osteoporosis** | 418 | - | 387 | - | 371 | - | 331 | - |
| None | 298 | 71.3 | 246 | 63.6 | 215 | 58.0 | 180 | 54.4 |
| Mild | 94 | 22.5 | 108 | 27.9 | 109 | 29.4 | 95 | 28.7 |
| Moderate | 18 | 4.3 | 27 | 7.0 | 38 | 10.2 | 46 | 13.9 |
| Strong | 8 | 1.9 | 6 | 1.6 | 9 | 2.4 | 10 | 3.0 |
| **Bone fracture** | 424 | - | 392 | - | 377 | - | 335 | - |
| No | 404 | 95.3 | 389 | 99.2 | 355 | 94.2 | 315 | 94.0 |
| Yes (accident) | 16 | 3.8 | 2 | 0.5 | 16 | 4.2 | 17 | 5.1 |
| Yes (without external impact) | 4 | 0.9 | 1 | 0.3 | 6 | 1.6 | 3 | 0.9 |
| **Hot flushes** | 422 | - | 398 | - | 376 | - | 333 | - |
| None | 164 | 38.9 | 116 | 29.1 | 83 | 22.1 | 67 | 20.1 |
| Mild | 119 | 28.2 | 107 | 26.9 | 111 | 29.5 | 91 | 27.3 |
| Moderate | 98 | 23.2 | 103 | 25.9 | 117 | 31.1 | 115 | 34.5 |
| Strong | 41 | 9.7 | 72 | 18.1 | 65 | 17.3 | 60 | 18.0 |
| **Relationship** | | | | | | | | |
| **Relationship** | 420 | - | 391 | - | 379 | - | 333 | - |
| No partner | 81 | 19.3 | 73 | 18.7 | 74 | 19.5 | 73 | 21.9 |
| Improved | 91 | 21.7 | 66 | 16.9 | 42 | 11.1 | 38 | 11.4 |
| Unchanged | 237 | 56.4 | 225 | 57.5 | 230 | 60.7 | 186 | 55.9 |
| Worsened | 11 | 2.6 | 27 | 6.9 | 33 | 8.7 | 36 | 10.8 |
| **Interest in sex** | 409 | - | 383 | - | 366 | - | 324 | - |
| Interest increased | 2 | 0.5 | 6 | 1.6 | 7 | 1.9 | 5 | 1.5 |
| Unchanged | 188 | 46.0 | 145 | 37.9 | 148 | 40.4 | 112 | 34.6 |
| Interest decreased | 142 | 34.7 | 144 | 37.6 | 131 | 35.8 | 113 | 34.9 |
| No interest at all | 77 | 18.8 | 88 | 23.0 | 80 | 21.9 | 94 | 29.0 |
