# Supplementary material for: Persistent impairments 3 years after (neo)adjuvant chemotherapy for breast cancer: results from the MaTox project
Source: Breast Cancer Res Treat. 2017 Jul 5;165(3):721–31. doi: 10.1007/s10549-017-4365-7 (PMC5602000; doi:10.1007/s10549-017-4365-7)
Supplement: Supplementary file 3 — Supplementary material 3 (PDF 166 kb) [file 10549_2017_4365_MOESM3_ESM.pdf]

Table S2. Patient and tumour characteristics – 3-year questionnaire yes/no

3-year questionnaire available	Yes (n=336)		No (n=117)	
	**Median**	**Min-Max**	**Median**	**Min-Max**
Age at diagnosis (years)	57	30-79	54	32-74
	**Mean**	**StD**	**Mean**	**StD**
BMI at enrolment	26.7	5.0	25.8	5.0
Patients with comorbidity [a]	**n**	**%**	**n**	**%**
Any comorbidity [b]	181	53.9	73	62.4
CCI =0 [c]	273	81.3	81	69.2
CCI ≥1[c]	63	18.8	36	30.8
Hypertension	99	29.5	31	26.5
Diabetes	23	6.8	13	11.1
Menopausal status [d]				
Premenopausal	89	26.5	38	32.5
Perimenopausal	16	4.8	2	1.7
Postmenopausal	199	59.2	61	52.1
Unknown	32	9.5	16	13.7
Receptor status [a]				
HR-positive, HER2-negative	181	53.9	56	47.9
HR-positive, HER2-positive	60	17.9	16	13.7
HR-negative, HER2-positive	31	9.2	7	6.0
Triple negative	54	16.1	32	27.4
Unknown	10	3.0	6	5.1
Tumour stage [a, e]				
I	92	27.4	26	22.2
II	151	44.9	50	42.7
III	49	14.6	22	18.8
Not determined/Unknown [f]	44	13.1	19	16.2
Nodal stage [a]				
Positive	162	48.2	60	51.3
Negative (N0)	166	49.4	51	43.6
Unknown (NX + missing)	8	2.4	6	5.2
Location of primary tumour [a]				
Right	163	48.5	69	59.0
Left	165	49.1	46	39.3
Both	8	2.4	2	1.7
Resection of primary tumour				
Breast-conserving (incl. follow-up resection)	235	69.9	64	54.7
Non-breast conserving (mastectomy/ablatio mammae)	88	26.2	42	35.9
Unknown	13	3.9	11	9.4
Radiotherapy received	265	78.9	89	76.1

Chemotherapy setting at enrolment*				
Neoadjuvant	29	8.6	29	24.8
Adjuvant	307	91.4	88	75.2
Taxane-based chemotherapy	194	57.7	81	69.2
Top chemotherapy regimen				
F+E/A+C±Tra	120	35.8	31	26.5
F+E/A+C+D±Tra	73	21.7	29	24.8
E/A+C+D±Tra	38	11.3	23	19.6
E/A+C+P±Tra	33	9.9	16	13.7
Car+D±Tra	13	3.9	5	4.3
Other	59	17.6	12	10.3
Endocrine therapy received	221	65.8	64	54.7
Endocrine therapy				
Aromatase inhibitors (AI) ±GnRH	94	28.0	26	22.2
Anti-oestrogen (AE)	71	21.1	25	21.4
Switch AI/AE	54	16.1	13	11.1
GnRH-analogue	2	0.6	-	-

[a] at diagnosis
[b] comorbidity according to Charlson [17] or additional concomitant diseases
[c] Charlson Comorbidity Index (CCI) according to Quan [18]
[d] at enrolment
[e] Tumour stage according to AJCC/UICC 7th edition
[f] for some patients the exact stage could not be determined because of unknown parameters (TX, NX, MX)
*Only patients who had received (neo)adjuvant chemotherapy were included in the present analysis, because the subgroup of patients who had received endocrine treatment only was too small for a meaningful analysis.

Abbreviations: AE, anti-oestrogen; AI, aromatase inhibitor; BMI, body mass index in kg/m²; C, cyclophosphamide; CCI, Charlson Comorbidity Index; Car, carboplatin; D, docetaxel; E/A, epirubicin/doxorubicin; F, fluorouracil; GnRH, gonadotropin-releasing hormone; HR, hormone receptor; HER2, human epidermal growth factor receptor 2; Max, maximum; Min, minimum; N, regional lymph node; P, paclitaxel; StD, standard deviation; Tra, trastuzumab.
