# Supplementary material for: Persistent impairments 3 years after (neo)adjuvant chemotherapy for breast cancer: results from the MaTox project
Source: Breast Cancer Res Treat. 2017 Jul 5;165(3):721–31. doi: 10.1007/s10549-017-4365-7 (PMC5602000; doi:10.1007/s10549-017-4365-7)
Supplement: Supplementary file 4 — Supplementary material 4 (PDF 176 kb) [file 10549_2017_4365_MOESM4_ESM.pdf]

Table S3 – Comparison PROs of the first questionnaire – 3-year questionnaire yes/no

Time point	4 weeks		4 weeks	
3-year questionnaire available	Yes		No	
	n	%	n	%
<u>**General condition**</u>				
**Tiredness**	311	-	105	-
None / never	31	10.0	16	15.2
Mild / rarely	140	45.0	43	41.0
Moderate / sometimes	117	37.6	39	37.1
Strong / often	23	7.4	7	6.7
Severe / always	0	-	0	-
**Irritability**	315	-	103	-
None	71	22.5	18	17.5
Mild	163	51.7	39	37.9
Moderate	73	23.2	41	39.8
Strong	8	2.5	5	4.9
**Despondence**	314	-	106	-
None	56	17.8	16	15.1
Mild	142	45.2	51	48.1
Moderate	91	29.0	31	29.2
Strong	25	8.0	8	7.5
**Exertion**	318	-	103	-
None	60	18.9	19	18.4
Mild	91	28.6	24	23.3
Moderate	96	30.2	38	36.9
Strong	71	22.3	22	21.4
<u>**Polyneuropathy symptoms**</u>				
**Numbness (fingers/toes)**	313	-	106	-
None / never	204	65.2	71	67.0
Mild / rarely	63	20.1	26	24.5
Moderate / sometimes	32	10.2	6	5.7
Strong / often	13	4.2	3	2.8
Severe / always	1	0.3	0	-
**Tingling / pain (fingers/toes)**	315	-	108	-
None	219	69.5	73	67.6
Mild	66	21.0	27	25.0
Moderate	26	8.3	7	6.5
Strong	4	1.3	1	0.9
**Weakness (arms/legs)**	316	-	106	-
None	189	59.8	58	54.7
Mild	86	27.2	34	32.1
Moderate	34	10.8	12	11.3
Strong	7	2.2	2	1.9

**<u>Post-surgical symptoms</u>**				
**Lymphedema**	302	-	95	-
None / never	207	68.5	60	63.2
Mild / rarely	59	19.5	21	22.1
Moderate / sometimes	28	9.3	9	9.5
Strong / often	7	2.3	5	5.3
Severe / always	1	0.3	0	-
**Pain (operated site)**	304	-	94	-
None	123	40.5	42	44.7
Mild	123	40.5	36	38.3
Moderate	50	16.4	12	12.8
Strong	8	2.6	4	4.3
**Impaired mobility**	303	-	93	-
None / never	129	42.6	37	39.8
Mild / rarely	116	38.3	33	35.5
Moderate / sometimes	39	12.9	15	16.1
Strong / often	19	6.3	7	7.5
Severe / always	0	-	1	1.1
**<u>Memory / attention</u>**				
**Impaired memory**	314	-	107	-
None / never	165	52.5	53	49.5
Mild / rarely	129	41.1	36	33.6
Moderate / sometimes	18	5.7	16	15.0
Strong / often	1	0.3	2	1.9
Severe / always	1	0.3	0	-
**Impaired concentration**	317	-	106	-
None / never	118	37.2	38	35.8
Mild / rarely	159	50.2	45	42.5
Moderate / sometimes	37	11.7	20	18.9
Strong / often	2	0.6	2	1.9
Severe / always	1	0.3	1	0.9
**<u>Appetite / smell / taste</u>**				
**Alterations in taste**	315	-	106	-
None	92	29.2	35	33.0
Mild	117	37.1	32	30.2
Moderate	93	29.5	36	34.0
Strong	13	4.1	3	2.8
**Appetite loss**	317	-	109	-
None / never	105	33.1	32	29.4
Mild / rarely	105	33.1	41	37.6
Moderate / sometimes	72	22.7	27	24.8
Strong / often	32	10.1	8	7.3
Severe / always	3	0.9	1	0.9

**Alterations in smell**	315	-	104	-
None	219	69.5	78	75.0
Mild	81	25.7	22	21.2
Moderate	13	4.1	3	2.9
Strong	2	0.6	1	1.0
**Cardiac function**				
**Respiratory distress**	315	-	108	-
None / never	152	48.3	51	47.2
Mild / rarely	119	37.8	42	38.9
Moderate / sometimes	36	11.4	12	11.1
Strong / often	7	2.2	3	2.8
Severe / always	1	0.3	0	-
**Heart problems**	317	-	107	-
None / never	216	68.1	71	66.4
Mild / rarely	82	25.9	28	26.2
Moderate / sometimes	16	5.0	5	4.7
Strong / often	3	0.9	3	2.8
Severe / always	0	-	0	-
**Fluid retentions/swollen limbs**	315	-	105	-
None / never	216	68.6	67	63.8
Mild / rarely	43	13.7	15	14.3
Moderate / sometimes	48	15.2	19	18.1
Strong / often	6	1.9	3	2.9
Severe / always	2	0.6	1	1.0
**Musculoskeletal system**				
**Pain joints**	315	-	107	-
None / never	93	29.5	31	29.0
Mild / rarely	146	46.3	51	47.7
Moderate / sometimes	60	19.0	19	17.8
Strong / often	13	4.1	6	5.6
Severe / always	3	1.0	0	-
**Pain muscles**	315	-	108	-
None	146	46.3	42	38.9
Mild	105	33.3	37	34.3
Moderate	54	17.1	19	17.6
Strong	10	3.2	10	9.3
**Pain spinal region**	315	-	104	-
None / never	80	25.4	31	29.8
Mild / rarely	134	42.5	36	34.6
Moderate / sometimes	71	22.5	27	26.0
Strong / often	26	8.3	10	9.6
Severe / always	4	1.3	0	-

**<u>Hormone-related symptoms</u>**				
**Osteoporosis**	313	-	105	-
None	224	71.6	74	70.5
Mild	68	21.7	26	24.8
Moderate	15	4.8	3	2.9
Strong	6	1.9	2	1.9
**Bone fracture**	317	-	107	-
No	300	94.6	104	97.2
Yes (accident)	13	4.1	3	2.8
Yes (without external impact)	4	1.3	0	-
**Hot flushes**	315	-	107	-
None	119	37.8	45	42.1
Mild	84	26.7	35	32.7
Moderate	78	24.8	20	18.7
Strong	34	10.8	7	6.5
**<u>Relationship</u>**				
**Relationship**	315	-	105	-
No partner	56	17.8	25	23.8
Improved	71	22.5	20	19.0
Unchanged	181	57.5	56	53.3
Worsened	7	2.2	4	3.8
**Interest in sex**	307	-	102	-
Interest increased	2	0.7	0	-
Unchanged	142	46.3	46	45.1
Interest decreased	108	35.2	34	33.3
No interest at all	55	17.9	22	21.6
